# Evaluation of Systemic Genotoxic/Oxidative and Proinflammatory Effects in Workers of a Titanium Dioxide Production Plant

**DOI:** 10.1155/2023/7066090

**Published:** 2023-07-22

**Authors:** Delia Cavallo, Anna Maria Fresegna, Aureliano Ciervo, Raffaele Maiello, Pieranna Chiarella, Giuliana Buresti, Valentina Del Frate, Marco Di Basilio, Sergio Iavicoli, Cinzia Lucia Ursini

**Affiliations:** ^1^Department of Occupational and Environmental Medicine, Epidemiology and Hygiene, INAIL, Monte Porzio Catone, Rome, Italy; ^2^Department of Technological Innovation and Safety of Plants, Products and Anthropic Settlements, INAIL, Monte Porzio Catone, Rome, Italy

## Abstract

This study is aimed at evaluating whether the occupational exposure to TiO_2_ during the industrial production process is able to induce genotoxic, oxidative, and inflammatory effects on blood, biomonitoring the same workers that showed micronucleus induction in the exfoliated buccal cells, as previous published. The final aim was to find sensitive and suitable biomarkers to evaluate potential early toxicity of occupational exposure to TiO_2_. On the same 40 workers involved in the manufacture of TiO_2_ pigment, 5 office workers, and 18 controls previously studied, we used formamidopyrimidine glycosylase- (Fpg-) comet assay on lymphocytes to evaluate genotoxic/oxidative effects and detected cytokine (IL-6, IL-8, and TNF*α*) release by ELISA to evaluate proinflammation. Moreover, we studied the possible influence of single nucleotide polymorphisms of XRCC1 and hOGG1 DNA repair genes and of GST metabolism-related genes (GSTT1 and GSTM1) on the evaluated effects. We did not find statistically significant differences in the mean values of the analysed Fpg-comet assay parameters; only the percentage of DNA damaged cells appearing in the test as comets (% comets) resulted higher in the exposed workers compared to controls. Also, the data analysed taking into account the specific task (bagging, industrial cleaning, mobile operations, maintaining, and production) showed differences only for % comets which resulted higher in industrial cleaners compared to controls. We found variations of IL-6 and IL-8 levels in the exposed workers with concentrations that were lower for IL-6 and higher for IL-8 compared to the control group. XRCC1, hOGG1, and GSTT1 polymorphisms did not influence neither comet parameters nor cytokine release. These findings demonstrate that TiO_2_ production process is able to induce slight proinflammatory effects in terms of IL-8 increased release but not significant genotoxic/oxidative effects on lymphocytes, which do not seem to be a target of TiO_2_, prevalently inhalable particles, generated in the studied production site.

## 1. Introduction

Titanium dioxide (TiO_2_) for its physicochemical characteristics is applied in a lot of products such as white colouring agent in paints, plastics, and papers and in food, cosmetics, and pharmaceuticals; TiO_2_ can be used as photocatalyst to decay environmental pollutants (http://www.tdma.info) [1]. In the world during 2019, the total amount of TiO_2_ produced was 6.300.000 tonnes [2]. The International Agency for Research on Cancer (IARC) in 2010 classified titanium dioxide (TiO_2_) as possibly carcinogenic for human (2B) based on sufficient evidence from experimental animals, specifically on the basis of two chronic inhalation studies in rats, and concluded that the evidence of carcinogenicity of TiO_2_ is inadequate in humans on the basis of three epidemiological cohort studies and one population-based case-control study from North America and Western Europe [3].

The largest of the epidemiological cohort studies was among male production workers in the TiO_2_ industry in six European countries and indicated a slightly increased risk for lung cancer compared with the general population with no evidence of an exposure-response relationship [4]. The other cohort studies, both conducted in the USA, did not report an increased risk for lung cancer or cancer at any other site [5, 6]. One population-based case-control study conducted in Montréal did not indicate an increased risk for lung or kidney cancer [7].

A review of the results of the experimental studies carried out *in vitro* and *in vivo* and published from 2013 to 2020, on genotoxicity of TiO_2_ on lung and intestinal models, demonstrates that TiO_2_ nano- and microparticles induce genotoxicity at concentrations similar to those reported by EFSA as realistic exposure scenario for food additive E171 [8]. In particular, EFSA estimated the exposure scenario ranging from 0.2 mg/kg b.w. per day for infants and the elderly to 5.5 mg/kg b.w. per day for children and 4 mg/kg b.w. per day for adults.

Chronic inhalation exposure to TiO_2_ can cause lung tumors in rats through a mechanism also relevant for other poorly soluble, low-toxicity particles including impaired clearance, inflammation, and regenerative epithelial cell proliferation [9]. According to the authors, a genotoxic mechanism of TiO_2_ action could occur but should be evaluated in pertinent tissues for exposure after inhalation and ingestion [9]. Their hypothesis is that rats could be more sensitive than humans to persistent inflammation and carcinogenicity induced in the lungs by TiO_2_.

Occupational exposure to TiO_2_ can happen during TiO_2_ production, packing, milling, or site cleaning by inhalation and ingestion [10]. Le et al. [11] reviewed the available studies in Europe and the United States on occupational exposure to TiO_2_, including the three epidemiological cohort studies considered by IARC, and did not find a clear evidence for correlation of TiO_2_ exposure and lung cancer.

According to the National Institute for Occupational Safety and Health (NIOSH), inhaled ultrafine TiO_2_ is a potential occupational carcinogen [12]. The threshold limit value (TLV) of the American Conference of Governmental Industrial Hygienists (ACGIH) for TiO_2_ FPs (total dust) is 10 mg/m^3^ as a time-weighted average (TWA) for a normal 8 h work day and a 40 h work week [13]. The recommended airborne exposure limits of NIOSH are 2.4 mg/m^3^ for fine (>100 nm) TiO_2_ particles and 0.3 mg/m^3^ for ultrafine (<100 nm, including engineered nanoscale) TiO_2_ particles, as time-weighted average (TWA) concentrations for up to 10 h/day during a 40-hour work week [12]. The Risk Assessment Committee (RAC) of European Chemicals Agency (ECHA) proposed in 2017 to classify TiO_2_ as a suspected carcinogen (category 2) by inhalation in certain powder forms under the CLP regulation [14], and the Commission Delegated Regulation (EU) 2020/217 of 4 October 2019 adopted this classification. On 18 February 2020, the note 10 was added, where TiO_2_ is classified as carcinogen by inhalation only for mixtures in powder form containing 1% or more of TiO_2_ in the form of, or incorporated in, particles with aerodynamic diameter ≤ 10 *μ*m [15]. In 2021, EFSA (European Food Safety Authority) updates the safety assessment of TiO_2_ used as food additive (E171) stating that “based on all the evidence available, a concern for genotoxicity could not be ruled out, and given the many uncertainties, E 171 can no longer be considered as safe when used as a food additive” [16]. In 2022, the Commission Delegated Regulation (EE) 2022/63 of 14 January 2022 amended the Annexes II and III of Regulation (EC) No. 1333/2008 of the European Parliament and of the Council as regards the food additive titanium dioxide (E 171) banning its use as food additive [17].

Recently, we published the first results of a biomonitoring study with Buccal Micronucleus Cytome (BMCyt) assay performed on workers involved in the production process of TiO_2_ particles where we found micronucleus induction in exfoliated buccal cells, demonstrating the suitability of this assay in the evaluation of early genotoxic and cytotoxic effects of TiO_2_ particles at the first contact site of inhalation exposure [18]. These workers were also simultaneously biomonitored detecting titanium and oxidized bases in urine after work shift by Buonaurio et al. who demonstrated the occurred exposure to TiO_2_ and the inducted oxidative stress [19].

In the present research, we aimed to evaluate whether the occupational exposure to TiO_2_, in the same production plant and on the same workers of the above cited studies, is able to induce genotoxic, oxidative, and inflammatory effects on blood. The final aims were to find good sensitive biomarkers to evaluate potential early toxicity of occupational exposure to TiO_2_ and to verify whether this kind of exposure has another target besides buccal cells. With this purpose, we chose Fpg-comet assay, which is a very sensitive biomarker of early genotoxic and oxidative effects. Moreover, since inflammation represents one of the key mechanisms of TiO_2_ toxicity [20], we also evaluated the potential proinflammatory effects of TiO_2_ occupational exposure detecting the IL-6, IL-8, and TNF*α* cytokine serum levels in workers and controls. In addition, since X-ray repair cross-complementing group 1 (XRCC1) gene polymorphism affects DNA repair efficiency and 8-oxoguanine DNA glycosylase-1 (h*OGG1*) *gene* plays a vital role in repairing oxidative DNA damage, we aimed to evaluate the possible influence of single nucleotide polymorphisms of XRCC1 and hOGG1 on the detected effects. Finally, since glutathione S-transferase (GST) genes encode enzymes mediating the detoxification of xenobiotics by catalyzing the conjugation of glutathione (GSH) to xenobiotic substrates and contribute to the protection from a broad range of compounds and oxidative stress products [21, 22], we evaluated the influence of GSTM1 and GSTT1 polymorphisms (with null genotype deficient in GST enzyme activity) on induced effects.

## 2. Material and Methods

### 2.1. Study Details

We previously described the studied production plant, where TiO_2_ pigment is manufactured from the titanium mineral ilmenite (iron and titanium oxide), using sulfate process and the results of personal monitoring of the exposure carried out on workers [18]. The latter reported a mean value of 0.16 mg/m^3^ for respirable particles and of 1.06 mg/m^3^ for inhalable particles and diameters in the range from 130 to 730 nm by SEM analysis of respirable particles collected on filters.

This research, representing the second part of Ursini et al.'s [18] study, was performed on the same workers involved in the steps from calcination to bagging (performed in the white area of industrial plant) and employed in five different tasks (maintainers, technicians, industrial cleaners, mobile operators, and bagging operators).

### 2.2. Subjects

All procedures of this study involving human participants were in accordance with the ethical standards of the Declaration of Helsinki. The study was conducted with the human subjects' understanding and consent. The enrolled subjects were 40 workers employed in the production of TiO_2_ (13 maintainers, 5 industrial cleaners, 9 mobile operators, 9 technicians, and 4 bagging operators) with a mean job seniority of 14 years and 5 workers employed in the administrative office of studied plant working near the production site (office workers). We selected, as group of control, 18 healthy subjects living in the same geographical area of studied workers and employed in administrative offices (external controls). We collected the data by a questionnaire including information regarding age, gender, smoking habits, job seniority, use of personal protective equipment, eating habits, scan tests, drug intake, and respiratory diseases.

### 2.3. Fpg-Comet Assay

Medical personnel collected whole venous blood samples in sterile heparinized vacutainer (BD Plymouth, UK) from enrolled subjects (exposed and control group) by venepuncture on Wednesday at the start-shift. Immediately after sample collection, all the samples were kept at 4°C and sent to the laboratory for further processing. We measured direct and oxidative DNA damage by formamidopyrimidine glycosylase- (Fpg-) modified Comet assay. Fpg is a bacterial enzyme that targets and cleaves oxidized purines, leaving a strand break detected by the comet assay as Fpg sites allowing to detect oxidative DNA damage. Peripheral blood lymphocytes were separated by density gradient with Lympholyte Cell Separation Media (human; Cedarlane, Burlington, Ontario, Canada) following the manufacturer's instructions. The samples were aliquoted and frozen in liquid nitrogen until they were used for DNA damage analysis. Unless otherwise stated, all materials and chemical reagents were from Sigma-Aldrich (currently Merck KGaA, Darmstadt, Germany). The procedure of Collins et al. [23], with minor modifications, was followed. At the time of use, the cell suspension of each subject was thawed and rinsed with PBS before being stratified on two GelBond Films (cut to the size of a glass microscope slide), one for the detection of direct DNA lesions (single- and double-strand breaks and alkali-labile sites) and one for oxidative DNA damage. Each GelBond Film was placed on a microscope slide. After lysis (2.5 M NaCl, 100 mM Na_2_EDTA, and 10 mM Tris with 1% Triton X-100 and 10% DMSO added fresh, pH 10, 1 h at 4°C in the dark), one set of slides was incubated with Fpg enzyme and the other one only with buffer (0.5 mM Na_2_EDTA, 0.1 M KCl, 40 mM HEPES-KOH, and 0.2 mg/ml BSA, pH 8, 30 min at 37°C in the dark). Then, the slides were placed in fresh alkaline buffer (1 mM Na_2_EDTA and 0.3 M NaOH, pH 13) in a horizontal electrophoresis tank for 40 min at 4°C to allow the denaturation and the unwinding of the DNA and the expression of alkaline-labile sites. Electrophoresis was performed in the same buffer at 25 V and 300 mA for 30 min to allow the fragments of damaged DNA to migrate towards the anode. The slides were then washed with Tris-HCl 0.4 M (pH 7.5) and stained with ethidium bromide (20 *μ*g/ml). The slides were examined at 200x magnification under a fluorescence microscope (Zeiss Axioplan 2 Imaging, Zeiss, Oberkochen, Germany). An undamaged cell appears as a nucleoid and a cell with damaged DNA as a comet. Images of at least 100 random comets from each sample were acquired (Zen 3 Blue Edition, Zeiss, Oberkochen, Germany) and analysed with a specific image analyser software (IAS, Delta Sistemi, Rome, Italy). For each subject, we calculated the mean values of the three main and more used parameters for indirect measurement of DNA damage: tail DNA%, tail length (TL), and tail moment (TM). Tail DNA% is the percentage of DNA in the tail of the comet; TL is the length of comet tail in micrometers, and TM, as the product of the TL and tail DNA%, provides an integrated measure of both of the above-mentioned parameters in arbitrary units. The combined use of these three parameters allows the estimation of the ability of a genotoxic agent to break the DNA strands into more or less small fragments. Therefore, to evaluate the direct DNA damage, we considered for each subject the mean value of the three parameters (tail DNA%, TM, and TL) of the Fpg enzyme-untreated cells.

We measured oxidative DNA damage by tail DNA%, following Collins' [24] suggestions, since it provides the best estimate of the frequency of DNA breaks, including those due to Fpg enzyme (relative to oxidized DNA bases). We used tail DNA% from Fpg enzyme-treated cells (tail DNAenz%, which evaluates direct and oxidative DNA damage) and subtracted tail DNA% from tail DNAenz% to obtain oxidative DNA damage (Fpg sites). Subjects with mean values of this difference (tail DNAenz%-tail DNA%) exceeding a fixed arbitrary cutoff value of 4 were considered positive for oxidative DNA damage, as previously reported by Cavallo et al. [25].

**Figure 1:**
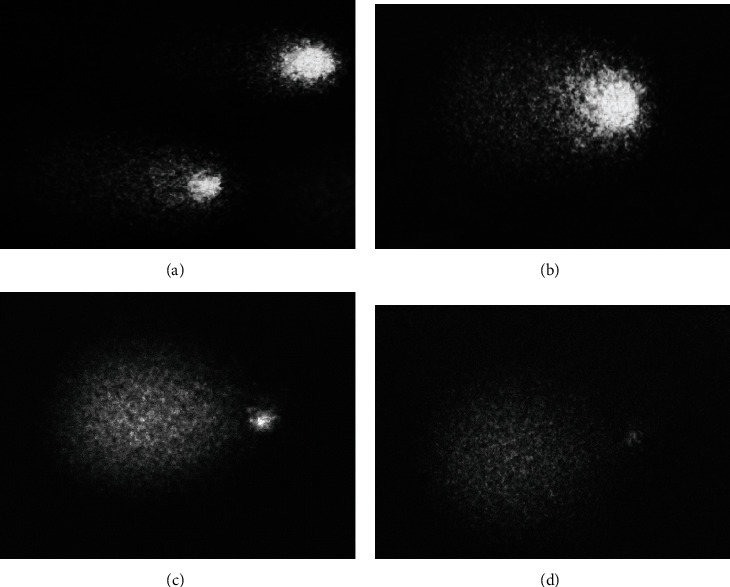
Comets (a, b) and apoptotic cells (c, d) by comet assay.

For each subject, we evaluated on a total of about 1000 cells of the Fpg-untreated sample: (1) the percentage of DNA damaged cells, appearing as comets corresponding to classes 2-3 of Collins' [24] classification, defined as “% comets” (Figures 1(a) and 1(b)) whose degree of DNA damage was evaluated by the parameters mentioned above (tail DNA%, TM, and TL), and (2) the percentage of extremely DNA damaged cells, appearing with the head of comet very small and the most of DNA in the tail (class 4 of Collins' [24] classification), defined as “% apoptotic cells” because they represent cells with nuclear condensation and DNA fragmentation characterizing the different stages of apoptosis (following Kulbacka et al.'s categorization [26]) (Figures 1(c) and 1(d)).

### 2.4. Fluorescence Microscopic Analysis of Apoptotic Cells

To confirm the apoptosis induction found by comet assay, an aliquot of frozen lymphocytes obtained from the selected subjects (those resulted by comet assay with % apoptotic cells higher than 1% and identified from “Exp A” to “Exp J” for the exposed workers and “Contr A” for the control) was defrosted, centrifuged, and fixed with a solution of methanol/acetic acid (purity 99.90%, Carlo Erba, Italy) (3 : 1 *v*/*v*) for 30 min. Then, the cells were centrifuged, washed twice in PBS, incubated for 15 min at 37°C with 0.5 *μ*g/ml Hoechst 33258 (Boehringer Mannheim, Germany) fluorescent dye, dropped onto slides, and covered with a coverslip (following Cavallo et al. [27] with minor modifications). Therefore, the cells were visualized for determination of nuclear chromatin morphology by fluorescence microscopy (Leica) at 1000x magnification. Apoptotic cells were recognized on the basis of nuclear condensation and/or fragmented chromatin. About 500 cells from each slide were examined for the presence of apoptotic features by an experienced observer, and % of total apoptotic cells was calculated (*n*apoptotic on 500 examined cells × 100).

### 2.5. Detection of Inflammatory Cytokine

TNF*α*, IL-6, and IL-8 cytokine releases were evaluated in the serum (stored at -80°C until use) of all the studied subjects by human enzyme-linked immunosorbent assay (ELISA) following the manufacturer's guidelines. We used eBioscience assay kits (eBioscience, Vienna, Austria) measuring the absorbance to 450 nm and quantified cytokine concentrations with a microplate reader (iMark, Bio-Rad, Segrate (Milano), Italy).

### 2.6. Polymerase Chain Reaction and Restriction Fragment Length Polymorphism

Whole blood of participants has been harvested by venipuncture to extract genomic DNA with the QiAamp DNA Blood Mini Kit (Qiagen, Germany, GmbH) following the manufacturer's instructions. The polymorphic genes analysed here have been already published in the scientific literature (see reference in Table 1). Standard PCR was carried out using 100 ng of human DNA, 1 unit of Taq polymerase enzyme (Promega, Madison, Wisconsin) per DNA sample (Applied Biosystems, Waltham, Massachusetts, United States), 0.3 *μ*M of forward and reverse primers, 0.2 mM of dNTP, and 2 mM of MgCl_2_. Synthetic oligonucleotides were purchased from Metabion GmbH International (AG Semmelweisstraße 3, 82152 Planegg, Germany) (Dasit Carlo Erba) and were shown below. Gene amplification of the investigated genes, using specific forward and reverse primers for each reaction, was assessed according to the methodology previously published (doi:10.5772/intechopen.86975). All PCR/digestion products of the investigated genes, shown in Table 1, were separated on 1% agarose gel (Euroclone S.p.A., Milan, Italy) with TBE buffer (Tris-borate-EDTA buffer) and stained with the gel red staining solution (Biotium, purchased directly to US-based customers). All gel images were visualized by automated chemiluminescence XRQ GeneSys imaging (gel documentation system, Syngene). In addition, since amplification conditions of both GST-T1 and GST-M1 have been shown as positive or null genotype, we added a positive control gene, i.e., the nuclear factor erythroid 2-related factor 2 (NRF2-653A/G) that was included in the PCR reaction. Amplification products were the following: GST-T1 458 bp, GSTM1 218 bp, and NRF2-653A/G 318 bp, the latter as control gene [28].

### 2.7. Statistical Methods

Statistical analysis was carried out by IBM SPSS software version 25. Both chi-square test and Fisher's exact test were considered to test the significance of the association between categorical variables and groups analysed.

After a preliminary test to verify the normality of parameter distributions (Shapiro-Wilk's test), in the case of Gaussian distribution, one-way ANOVA and Student's *t*-test were considered to test the significance of mean value differences between controls and exposed subgroups/group. When non-Gaussian distribution was confirmed, the Kruskal-Wallis and Mann-Whitney nonparametric tests were considered in the analysis. We applied Dunn's procedure with a Bonferroni correction for multiple comparisons to determine whether differences exist among the means of groups.

The multiple regression analysis was performed using the studied biomarkers of effects as the dependent variables and the exposure, confounding factors, and polymorphisms as the independent variables. A *p* value < 0.05 was considered significant.

## 3. Results

Control subjects and potentially exposed subjects did not show statistically significant differences relatively to smoking habits, age (41.55 ± 8.86 years for exposed workers in respect to 40.33 ± 12.50 for external controls), and job seniority (14.31 ± 9.71 years for exposed workers) [18]. Regarding gender, more than 90% of exposed workers and external controls were males in respect to the 40% of the office workers. Tables 2 and 3 describe additional confounding factors as eating habits, scan tests, and drug intake relative to the studied subjects, and they did not show statistically significant differences.  Table 3 also shows the percentages of subjects who declared in the questionnaire to be suffering from respiratory diseases, prevalently bronchial asthma, either taking into account all the enrolled subjects or whether they produced TiO_2_ or not. The data show that the percentage of TiO_2_-producing workers who declared to be affected by respiratory diseases, prevalently bronchial asthma, was higher than in the external controls (20.5% vs. 5.6%), even if the difference was not statistically significant.

Table 4 shows the results of the Fpg-comet assay and cytokine detection obtained on workers employed in the production of TiO_2_, office workers, and controls. Fpg-comet assay demonstrates that, regarding the direct DNA damage parameters, there were no statistically significant differences among the three analysed groups, whereas mean value of comet percentage (% comets) was higher (*p* = 0.049) in the group employed in the TiO_2_ production in comparison with the external control group. We found higher percentages of subjects positive to oxidative DNA damage in the workers producing TiO_2_ and in office workers in respect to external controls; however, the differences were not statistically significant.

Regarding the detection of IL-6, we found lower concentrations in the exposed “TiO_2_ production” workers and “office workers” in comparison with the external control group. Differently, IL-8 levels were higher in the exposed “TiO_2_ production” workers in respect to the control group.

Also when we took into consideration the specific tasks of the enrolled subjects (Tables 5 and 6), the Fpg-comet assay did not show statistically significant differences in the mean values of tail DNA%, TM, and TL whereas mean value of % comets resulted higher (*p* = 0.013) in the industrial cleaner group compared to external controls. The highest percentage of apoptotic cells (% apoptotic cells) was found in the maintainer group, but the difference was not statistically significant. The maintainer group also resulted the group with the highest percentage of positive subjects to oxidative DNA damage followed by bagging operators. Oxidative DNA damage in terms of Fpg site mean values higher than cutoff = 4 was found in bagging operators and technicians.

To confirm the apoptosis results obtained by comet assay, we also performed the fluorescence microscopic analysis as additional test in those subjects resulted with percentages of apoptotic cells higher than 1%. The results are reported in Table 7 and show similar values.

In Table 8, we show the results of cytokine detection analysed taking into account the specific task of the exposed workers. IL-6 release was in industrial cleaners, mobile operators, and maintainers statistically significantly lower than in controls whereas in bagging operators and technicians resulted higher than in controls even if the difference was not statistically significant. Other differences were found between the different tasks. Among the specific analysed tasks, technicians showed the highest mean value of IL-8 concentration that was statistically significant compared with the controls; also, bagging operators showed higher IL-8 release than controls not reaching however the statistical significance. The slight oxidative and proinflammatory effects found in both bagging operators and technicians could be related with the specific tasks involved in the last phases of the production process when final small-sized TiO_2_-produced material was handled.

Table 9 shows the results of the comparison of the distribution of hOGG1, XRCC1, and GST genotypes in the groups and demonstrates that the percentages of subjects mutant+heterozygous (*mut+het*) for hOGG1 and XRCC1 are comparable with those wild type (*wt*) and that the percentage of subjects *null* for GSTT1 is comparable with those *positive* (*pos*) either in subjects taken all together or in workers producing TiO_2_ and in controls, without significant differences between exposed workers and controls. While for GSTM1, the percentage of subject with *null* genotype was significantly higher than those with *positive* genotype (GSTM1 *pos*) in exposed subjects unlike controls, with significant differences between exposed workers and controls. The analysis of hOGG1 gene polymorphism has been possible only on 43 subjects, including 22 workers producing TiO_2_, 3 office workers, and 18 controls. In particular, the comparison performed (by the Mann-Whitney test) on mean values of Fpg-comet results and cytokine release of *mut+het* subjects compared to *wt* subjects of each group, to evaluate the possible influence of this gene polymorphism, did not find any statistically significant difference. However, we observed in all the studied populations a higher oxidative DNA damage mean value in *mut+het* subjects (characterized by reduced efficiency of 8-oxoguanine DNA glycosylase) in respect to *wt* subjects. The analysis performed on all the enrolled subjects for XRCC1 gene did not find any influence on Fpg-comet parameters nor for cytokine release. Relatively to the possible influence of GSTT1 polymorphism on mean values of Fpg-comet results and cytokine release, we did not find any statistically significant difference between GSTT1 *pos* and *null* subjects. We did not analysed the possible influence of GSTM1 because there was a statistically significant different frequency distribution of *null/pos* genotypes between exposed workers and controls. We also analysed the possible association (by Fisher's test and chi-square test) of positivity to oxidative DNA damage with the OGG1, XRCC1, GSTT1, and GSTM1 polymorphisms, but we did not find any statistically significant association.

The regression analysis performed to evaluate the influence of confounding factors (such as smoking habits and age), gene polymorphisms, and exposure, used as independent variables, on comet percentage and apoptotic cells did not result to be influenced by any of the analysed independent variables.

## 4. Discussion

The present investigation focused on the very sensitive biomarker Fpg-comet assay which is able to detect early direct and oxidative DNA damage, because several *in vitro* and *in vivo* studies demonstrated genotoxic effects of TiO_2_ using this assay [8] and on the detection of proinflammatory IL-6, IL-8, and TNF*α*, since inflammation represents one of the key mechanisms of toxicity of TiO_2_ [20]. On the same workers enrolled in our first study [18], we performed Fpg-comet assay on lymphocytes obtained by peripheral blood collected simultaneously to buccal cells. The present study demonstrates that Fpg-comet assay on lymphocytes did not detect statistically significant direct or oxidative DNA damage in the tested workers involved in the production process of TiO_2_ particles although this assay found higher comet percentages and higher percentage of subjects positive to oxidative DNA damage, in workers producing TiO_2_ compared to controls.

The comparison between the lack of genotoxicity found by the Fpg-comet assay parameters (TM, TL, and tail DNA%), able to detect direct DNA damage, and the induction of MN in buccal cells, found by BMCyt assay in the biomonitoring of the same workers previously published [18], seems contrasting. These different results could be explained by the difference in the biological matrices, and buccal cells represent a direct target of exposure by inhalation, whereas probably lymphocytes are more difficult to be reached by the particles, particularly nonrespirable particles (>4 *μ*m). Moreover, buccal cells have lower capacity to repair DNA compared to peripheral blood lymphocytes because DNA damage in the mitotic basal layer of the epithelial tissue cannot be repaired in the process of differentiation; therefore, these cells provide a more reliable measure of genomic instability events in the epithelial respiratory tract [29]. In addition, the genotoxic effects detected by the comet assay and MN tests are due to different mechanisms; the MN test detects damages that survive at least one mitotic cycle, while the comet assay identifies repairable DNA strand breaks or alkali-labile sites, so the use of both the MN test and the comet assay is advisable [30].

We also found in the exposed subject modifications in the cytokine IL-6 and IL-8 releases in comparison with controls, with different trends, decreasing for IL-6 levels (industrial cleaners, mobile operators, and maintainers) and increasing for IL-8 (technicians and in lower extend bagging operators). Cytokine decrease was already shown in workers with long-term exposure to diesel exhaust engine in the study of Dai et al. [31] that, according to the authors, could be associated to immune pathological injury. Also in our study, the TiO_2_-producing workers were long-term exposed; in fact, the mean value of job seniority is 14 years; therefore, we could make the same hypothesis of Dai et al. [31]. The Katiyar et al. [32] study demonstrated that IL-6 levels were suppressed in the chromium-exposed groups as compared to unexposed healthy volunteers, suggesting that lower blood IL-6 could be harmful because it could make chromium-exposed workers more prone to infections. When we took into account the specific task, we found statistically significant decrease of IL-6 levels in maintainers, industrial cleaners, and mobile operators. The maintainers and industrial cleaners were also the groups with the highest frequencies of micronucleus and condensed chromatin on buccal cells in our previous study performed on the same workers [18]. This suggests a possible correlation between buccal cytogenotoxic effect and decrease of IL-6 in the exposure to TiO_2_, but this result needs to be confirmed by further studies.

The slight but statistically significant increase of IL-8 cytokine release found in technicians, involved in the production process management, and the increase found in bagging operators (involved in bagging of produced TiO_2_) together with an increase, even if not significant, found in both these tasks also for IL-6, seem to confirm the potential inflammatory effect of TiO_2_ [33–35]. Bagging operators and technicians showed also oxidative DNA damage in terms of Fpg site mean values, slightly higher than cutoff suggesting possible oxidative effects. These results need to be confirmed on a higher number of subjects involved in the same task to clarify the induced effect and the possible relative correlation. However, a higher percentage of subjects with respiratory diseases, prevalently bronchial asthma, was found in exposed workers in comparison with controls, even if the difference was not statistically significant, suggesting a possible association with TiO_2_ exposure.

Regarding the evaluation of the influence of the analysed polymorphisms on the observed effects, the analysis of possible influence of hOGG1 polymorphism on the genotoxic effects did not find any influence. Also, the evaluation of the possible influence of single nucleotide polymorphisms of XRCC1 (known to affect DNA repair efficiency) on the damage induced by occupational TiO_2_ exposure did not show any influence.

## 5. Conclusions

In conclusion, the present study demonstrates that TiO_2_ production process, in the studied plant, does not induce significant systemic genotoxic/oxidative effects. The exposure was able to induce only slight but significant IL-8 increased release in technicians and at lower extend in bagging operators. TiO_2_ exposure slightly reduced IL-6 release in other tasks suggesting a possible immune pathological injuring after long exposure, which needs yet to be confirmed on a higher number of subjects. In technicians and bagging operators, we also found a slight oxidative DNA damage increase in terms of Fpg sites that could be related to the slight proinflammatory effects shown in these specific tasks (involved in the last phases of the production process when final small-sized TiO_2_-produced material was handled).

The comparison of the findings of the present study with those previously obtained on the buccal cells of the same workers demonstrates that only buccal cells, the first contact site for inhalable particles, are damaged after TiO_2_ occupational exposure, and therefore, they represent a more suitable biological matrix for the biomonitoring of workers producing TiO_2_ particles with different sizes, also considering that they are obtained in a noninvasive way, whereas for the TiO_2_ particles produced in the studied plant, as prevalently inhalable particles not penetrating deeply in respiratory system, lymphocytes do not seem to be a target of genotoxic effect.

## Figures and Tables

**Table 1:** Analysis of human polymorphic genes in the Caucasian population.

Gene polymorphism	rs number	Primer sequence	Annealing temperature	Restriction enzyme pattern	Results	Reference
GST-T1	rs17856199	F: 5′-TTC CTT ACT GGT CCT CAC ATC TC-3′	62°C	Absence of restriction pattern	Positive/null	Teixeira, J.P., et al. Toxicology. 2004; 195 (2-3), 231-242
		R: 5′-TCA CCG GAT CAT GGC CAG CA-3′				

GST-M1	rs366631	F: 5′-GAA CTC CCT GAA AAG CTA AAG C-3′	62°C	Absence of restriction pattern	Positive/null	Sørensen M. et al. Int J Cancer. 2004; 110(2):219-224
		R: 5′-GTT GGG CTC AAA TAT ACG GTG G-3′				

hOGG1 326	rs1052133	F: 5′-GGA AGG TGC TTG GGG AAT-3′	58°C	Fnu4HI	Wild type: 200 bp; heterozygous: 200 bp, 100 bp; mutant: 100 bp	Le Marchand et al. Cancer Epidemiology, Biomarkers and Prevention. 2002; 11 (4):409-412
		R: 5′-ACT GTC ACT AGT CTC ACC AG-3′				

XRCC1 399 G/A	rs 25487	F: 5′-TTG TGC TTT CTC TGT GTC CA-3′	62°C	MSPI	Wild type: 374 and 221 bp; heterozygous: 615 bp, 374 and 221 bp; mutant: 615 bp	Kowalski et al. Journal of Experimental and Clinical Cancer Research. 2009; 28 : 37
		R: 5′-TCC TCC AGC CTT TTC TGA TA-3′				

XRCC1: X-ray repair cross-complementing group 1; hOGG1: 8-oxoguanine DNA glycosylase-1; GST: glutathione S-transferase.

**Table 2:** Dietary habits in the studied populations.

Category	Total *N* = 63		All workers employed in TiO_2_ plant *N* = 45		Workers producing TiO_2_*N* = 40		Office workers *N* = 5		External controls *N* = 18		*p* value
	*N*	%	*N*	%	*N*	%	*N*	%	*N*	%	
Fruit consumption											
*Never*	1	1.6	0	0.0	0	0.0	0	0.0	1	5.6	(a) *χ*^2^ = 0.047
*Rarely*	6	9.5	2	4.4	2	5.0	0	0.0	4	22.2	(b) *χ*^2^ = 0.103
*Daily basis*	26	41.3	21	46.7	17	42.5	4	80.0	5	27.8	
*Many times a day*	30	47.6	22	48.9	21	52.5	1	20.0	8	44.4	
Vegetable consumption											
*Never*	1	1.6	1	2.2	1	2.5	0	0.0	0	0.0	(a) *χ*^2^ = 0.308
*Rarely*	7	11.1	3	6.7	3	7.5	0	0.0	4	22.2	(b) *χ*^2^ = 0.625
*Daily basis*	29	46.0	21	46.7	19	47.5	2	40.0	8	44.4	
*Many times a day*	26	41.3	20	44.4	17	42.5	3	60.0	6	33.3	
Grilled fish/meat consumption											
Never	7	11.5	5	11.4	4	10.3	1	20.0	2	11.8	(a) *χ*^2^ = 0.469
Once a month	27	44.3	22	50.0	20	51.3	2	40.0	5	29.4	(b) *χ*^2^ = 0.437
Two or three times a month	15	24.6	10	22.7	10	25.6	0	0.0	5	29.4	
More than 3 times a month	12	19.7	7	15.9	5	12.8	2	40.0	5	29.4	

(a) Comparison among exposed and controls. (b) Comparison among kind of exposure and controls.

**Table 3:** X-ray, drug intake, and diseases in the studied populations.

	Total *N* = 63		All workers employed in TiO_2_ plant *N* = 45		Workers producing TiO_2_*N* = 40		Office workers *N* = 5		External controls *N* = 18		*p* value
	*N*	*%*	*N*	%	*N*	%	*N*	%	*N*	%	
Scan test (12 months)											
Yes	11	17.5	6	13.3	5	12.5	1	20.0	5	27.8	(a) *χ*^2^ = 0.172
No	52	82.5	39	86.7	35	87.5	4	80.0	13	72.2	(b) *χ*^2^ = 0.362
Drug (3 months)											
Yes	36	83.7	25	89.3	20	87.0	5	100.0	11	73.3	(a) *χ*^2^ = 0.177
No	7	16.3	3	10.7	3	13.0	0	0.0	4	26.7	(b) *χ*^2^ = 0.311
Respiratory diseases											
Yes	9	14.5	8	18.2	8	20.5	0	0.0	1	5.6	(a) *χ*^2^ = 0.200
No	53	85.5	36	81.8	31	79.5	5	100.0	17	94.4	(b) *χ*^2^ = 0.208

(a) Comparison among exposed and controls. (b) Comparison among kind of exposure and controls.

**Table 4:** Fpg-comet parameters and cytokine release in the studied populations.

Biomarkers	Type of exposure	*N*	Mean	SD	*p* value	Post hoc (Dunn's test)^∗^	Adj. *p* values
% DNA tail	TiO_2_ production	40	12.78	2.52	0.088 (Kruskal-Wallis)		
	Office workers	5	14.08	2.24			
	Ext controls	18	11.56	2.62			

TM	TiO_2_ production	40	3.09	0.78	0.164 (Kruskal-Wallis)		
	Office workers	5	3.78	0.88			
	Ext controls	18	2.92	1.02			

TL	TiO_2_ production	40	16.44	6.09	0.060 (Kruskal-Wallis)		
	Office workers	5	25.51	6.14			
	Ext controls	18	17.65	8.33			

% comets	TiO_2_ production	40	19.78	1.61	0.048 (Kruskal-Wallis)	TiO_2_ production > controls	0.049
	Office workers	5	19.00	0.35			
	Ext controls	18	18.86	1.55			

% apoptotic cells	TiO_2_ production	40	0.88	0.43	0.126 (ANOVA)		
	Office workers	5	0.70	0.16			
	Ext controls	18	0.65	0.36			

Oxidative DNA damage (Fpg sites)	TiO_2_ production	40	3.34	3.33	0.206 (ANOVA)		
	Office workers	5	5.01	2.24			
	Ext controls	18	2.47	1.60			

Oxidative positive subjects > cutoff 4	TiO_2_ production	14/40 (35.0%)			0.570 (Fisher's exact test)		
	Office workers	2/5 (40.0%)					
	Ext controls	4/18 (22.2%)					

IL6 (pg/ml)	TiO_2_ production	40	0.24	0.33	Kruskal − Wallis < 0.001	TiO_2_ production < controls	0.001
	Office workers	5	0.00	0.00		Office workers < controls	0.003
	Ext controls	18	0.48	0.18			

IL8 (pg/ml)	TiO_2_ production	40	1.88	1.63	0.006 (Kruskal-Wallis)	TiO_2_ production > controls	0.004
	Office workers	5	1.46	1.86			
	Ext controls	18	1.23	2.71			

TNF*α* (pg/ml)	TiO_2_ production	40	0.72	2.06	0.538 (Kruskal-Wallis)		
	Office workers	5	0.00	0.00			
	Ext controls	18	0.31	1.14			

^∗^Pairwise comparisons were performed using Dunn's procedure with a Bonferroni correction for multiple comparisons. Adjusted *p* values are presented.

**Table 5:** Fpg-comet results: direct DNA damage analysed taking into account the specific task.

Parameters/task	*N*	Mean	SD	*p* value
*Tail DNA%*				
Bagging operators	4	12.58	0.74	0.256 (ANOVA)
Industrial cleaners	5	13.90	4.74	
Mobile operators	9	11.65	1.83	
Maintainers	13	13.23	2.51	
Technicians	9	12.72	2.05	
Office workers	5	14.08	2.24	
External controls	18	11.56	2.62	
*TM*				
Bagging operators	4	3.30	0.69	0.166 (ANOVA)
Industrial cleaners	5	3.55	0.97	
Mobile operators	9	2.56	0.70	
Maintainers	13	3.09	0.65	
Technicians	9	3.26	0.83	
Office workers	5	3.78	0.88	
External controls	18	2.92	1.02	
*TL*				
Bagging operators	4	19.51	8.06	0.139 (Kruskal-Wallis)
Industrial cleaners	5	12.29	3.23	
Mobile operators	9	14.90	5.56	
Maintainers	13	17.50	6.32	
Technicians	9	17.40	6.28	
Office workers	5	25.51	6.14	
External controls	18	17.65	8.33	

**Table 6:** Fpg-comet results: comet percentages, apoptotic cells, and oxidative DNA damage analysed taking into account the specific task.

Markers/task	*N*	Mean	SD	*p* value	Multiple comparisons	
*% comets*						
Bagging operators	4	18.88	1.26	0.026 (ANOVA)	Post hoc (Bonferroni test)	*p* value
Industrial cleaners	5	21.56	2.65			
Mobile operators	9	19.26	0.65			
Maintainers	13	19.61	1.05		Industrial cleaners < control	0.013
Technicians	9	19.97	1.94			
Office workers	5	19.00	0.35			
External controls	18	18.86	1.55			
*% apoptotic cells*						
Bagging operators	4	0.85	0.39	0.129 (ANOVA)		
Industrial cleaners	5	0.76	0.65	
Mobile operators	9	0.64	0.23	
Maintainers	13	1.03	0.44	
Technicians	9	0.96	0.42	
Office workers	5	0.70	0.16	
External controls	18	0.65	0.36	
*Oxidative DNA damage*						
Bagging operators	4	4.10	0.55	0.361 (Kruskal-Wallis)		
Industrial cleaners	5	3.48	4.00	
Mobile operators	9	2.98	1.38	
Maintainers	13	2.75	4.25	
Technicians	9	4.12	3.91	
Office workers	5	5.01	2.24	
External controls	18	2.47	1.60	
*Oxidative positive subjects* > cutoff 4						
Bagging operators	2/4 (50%)			0.493 (Fisher's exact test)		
Industrial cleaners	1/5 (20%)			
Mobile operators	2/9 (22.2%)			
Maintainers	7/13 (53.8%)			
Technicians	2/9 (22.2%)			
Office workers	2/5 (40%)			
External controls	4/18 (22.2%)			

**Table 7:** Comparison between comet assay and fluorescence microscopy results of apoptosis evaluation in the selected subjects (with comet % apoptotic cells > 1%).

Subject code	Task	Comet assay % apoptotic cells	Microscopic analysis % apoptotic cells
Exp A	Bagging operator	1.2	1.1
Exp B	Industrial cleaner	1.5	1.5
Exp C	Maintainer	1.3	1.3
Exp D	Maintainer	1.1	1.2
Exp E	Maintainer	1.8	1.5
Exp F	Maintainer	1.7	1.5
Exp G	Maintainer	1.1	1.2
Exp H	Technician	1.9	1.6
Exp I	Technician	1.2	1.5
Exp J	Technician	1.1	1.0
		1.39 ± 0.31 (mean value ± SD)	1.34 ± 0.20 (mean value ± SD)
Contr A		1.4	1.5

**Table 8:** Cytokine detection results analysed taking into account the specific task.

Markers/task	*N*	Mean	SD	*p* value	Multiple comparisons	
					Post hoc (Dunn's test)^∗^	Adjusted *p* values
*IL-6 (pg/ml)*						
Bagging operators	4	0.58	0.23	K‐W < 0.001	Maintainers < technicians	0.001
Industrial cleaners	5	0.01	0.02		Office workers < technicians	0.002
Mobile operators	9	0.08	0.18		Mobile operators < technicians	0.005
Maintainers	13	0.05	0.08		Industrial cleaners < technicians	0.006
Technicians	9	0.66	0.31		Office workers < bagging operators	0.046
Office workers	5	0.00	0.00		Industrial cleaners < controls	0.014
External controls	18	0.48	0.18		Mobile operators < controls	0.008
					Maintainers < controls	0.001
					Office workers < controls	0.005

*IL-8 (pg/ml)*						
Bagging operators	4	2.71	1.18	0.002 (K-W)		
Industrial cleaners	5	0.77	1.04		Post hoc (Dunn's test)^∗^	Adj. *p* values
Mobile operators	9	1.02	0.89			
Maintainers	13	1.72	1.84		Technicians > controls	0.001
Technicians	9	3.20	1.46			
Office workers	5	1.46	1.86			
External controls	18	1.23	2.71			

*TNFα (pg/ml)*						
Bagging operators	4	2.70	3.89	0.016 (K-W)	Post hoc did not reveal any significance among the tasks	
Industrial cleaners	5	0.00	0.00			
Mobile operators	9	0.00	0.00			
Maintainers	13	0.73	2.65			
Technicians	9	0.95	1.23			
Office workers	5	0.00	0.00			
External controls	18	0.31	1.14			

^∗^Pairwise comparisons were performed using Dunn's procedure with a Bonferroni correction for multiple comparisons. Adjusted *p* values are presented.

**Table 9:** hOGG1, XRCC1, and GST genotype distribution in exposed workers and controls.

	All subjects		Workers producing TiO_2_		Office workers		External controls		*p* value
	*N*	%	*N*	%	*N*	%	*N*	%	
hOGG1									
*mut+het*	15	34.9	7	31.8	1	33.3	7	38.9	Fisher's exact test = 0.750
*wt*	28	65.1	15	68.2	2	66.7	11	61.1	
XRCC1									
*mut+het*	36	57.1	20	50.0	4	80,0	12	66.7	Chi-square test (*χ*^2^) 0.334
*wt*	27	42.9	20	50.0	1	20.0	6	33.3	
GSTT1									
*pos*	54	85.7	34	85.0	4	80.0	16	88.9	Fisher's exact test = 0.868
*null*	9	14.3	6	15	1	20.0	2	11.1	
GSTM1									
*pos*	26	41.3	11	27.5	2	40.0	13	72.2	Fisher's exact test^∗^ = 0.004
*null*	37	58.7	39	72.5	3	60.0	5	27.8	

^∗^ indicates statistically significant.

## Data Availability

All relevant data are included in the manuscript. Further inquiries may be directed to the corresponding author (d.cavallo@inail.it).
